# Rapid multiplex gene expression assays for monitoring metabolic resistance in the major malaria vector *Anopheles gambiae*

**DOI:** 10.1186/s13071-018-3253-2

**Published:** 2019-01-06

**Authors:** Konstantinos Mavridis, Nadja Wipf, Sandrine Medves, Ignacio Erquiaga, Pie Müller, John Vontas

**Affiliations:** 1 Institute of Molecular Biology and Biotechnology, Foundation for Research and Technology-Hellas, 70013 Heraklion, Greece; 2 Department of Epidemiology and Public Health, Swiss Tropical and Public Health Institute, Socinstrasse 57, P.O. Box, CH-4002 Basel, Switzerland; 3 University of Basel, Petersplatz 1, P.O. Box, CH-4001 Basel, Switzerland; 4 Fast Track Diagnostics, a Siemens Healthineers Company, Esch-sur-Alzette, 4354 Luxembourg; 5 Pesticide Science Laboratory, Department of Crop Science, Agricultural University of Athens, 11855 Athens, Greece

**Keywords:** Multiplex TaqMan assays, Detoxification, P450s, GSTEs, Insecticide resistance, Metabolic resistance, Gene expression

## Abstract

**Background:**

Metabolic resistance of the major malaria vector *Anopheles gambiae* (*s.l.*) to insecticides is operationally significant, particularly in combination with target site resistance. However, detection of metabolic resistance is not trivial and relies on laborious bioassays, unspecific biochemical methods, or sophisticated and expensive molecular approaches using transcriptomics.

**Methods:**

Rapid one-step multiplex TaqMan-probe based RT-qPCR assays were developed and optimised to measure the expression levels of genes associated with metabolic insecticide resistance in *An. gambiae* (*s.l.*). Primers and probes were designed to target the mRNA of cytochrome P450-dependent monooxygenases *CYP6P3*, *CYP6M2*, *CYP9K1*, *CYP6P4* and *CYP6Z1*, and the glutathione-S-transferase *GSTE2*. The novel assays were validated *versus* gold standard methods with a range of phenotyped mosquito specimens. The assays were also tested directly on lysates of RNA*later*®-preserved mosquitoes without an RNA extraction step.

**Results:**

The novel assays are efficient (reaction efficiencies = 95–109%), sensitive (covering a > 10.0 Ct range with R^2^ values > 0.99), specific (TaqMan chemistry), reproducible (%CV = 4.46–12.07%), as well as readily expandable to capture additional loci as they evolve or to cover additional species. The assays were successfully validated in terms of expression levels against standard two-step singleplex qPCR assays (overall % difference = -17.6%, 95% CI = -38.7–3.43%) and microarrays, using laboratory strains and field-caught samples. The assays can also be applied directly on lysates of mosquito specimens, without RNA extraction or DNase treatment.

**Conclusions:**

The novel multiplex assays for monitoring the levels of major detoxification genes and metabolic resistance in *An. gambiae* (*s.l.*) are simple to perform, robust and rapid. They may complement current diagnostic assays to provide evidence-based and operationally relevant information for insecticide resistance management.

**Electronic supplementary material:**

The online version of this article (10.1186/s13071-018-3253-2) contains supplementary material, which is available to authorized users.

## Background

Insecticide based vector control interventions have reduced malaria incidence [1]. However, the increasing use of a limited number of insecticides, primarily pyrethroids, places an immense selection pressure on insect populations, which has not left disease vectors unaffected [2, 3]. The resulting insecticide resistance (IR) in the major malaria vector *Anopheles gambiae* (*s.l*.) represents one of the greatest challenges in malaria control. In *Anopheles* mosquitoes, resistance is primarily conferred by mutations at the insecticide’s target site that alter its sensitivity, and by the upregulation of enzymes that detoxify or sequester the insecticide [4]. Several cytochrome P450-dependent monooxygenases have been functionally associated with pyrethroid resistance. CYP6P3 [5] and CYP6M2 [6] are considered as the main pyrethroid metabolising enzymes in several *An. gambiae* populations in West Africa. *CYP6Z1* is associated with both pyrethroid and dichlordiphenyltrichlorethan (DDT) resistance [7]. *CYP6P4* is associated with resistance to both alpha-cyano and non-alpha-cyano pyrethroids [8], and metabolises the juvenile hormone pyriproxyfen that is used as insect growth regulator by preventing larvae from developing into adult stages [9]. *CYP9K1* was recently found to be overexpressed in deltamethrin-resistant Bioko populations and also metabolises pyrethroids [10]. The glutathione-S-transferase *GSTE2* is associated with DDT [11] and pyrethroid [12] resistance.

Up-to-date data on resistance to insecticides are a prerequisite for the effective implementation of interventions. While a multitude of high throughput assays for the detection of individual target site resistance mutations have been developed and used to facilitate the implementation of vector control strategies [13, 14], there are very few such tools available for monitoring metabolic insecticide resistance in mosquito field populations [12]. In the absence of DNA markers associated with overexpression of detoxification genes for *An. gambiae* (*s.l.*), detection methods for metabolic resistance are restricted to either phenotypic bioassays with synergists or biochemical assays. Synergists are compounds that inhibit metabolic detoxification enzymes. By comparing the mortality of mosquitoes exposed to a synergist followed by the insecticide to the mortality of mosquitoes exposed to only the insecticide, a conclusion can be drawn as to whether and which enzyme families are involved in the observed resistance [15]. Two major drawbacks of synergist-insecticide bioassays are that they require large numbers of live mosquitoes and that they may only indicate which enzyme class (e.g. P450s, GSTs or esterases) is implicated and not specify which one of the enzymes is overexpressed. Biochemical measurement of resistance enzyme activity on microplates represents a simpler alternative to phenotypic response-to-exposure tests [16]. However, the biochemical assays’ generic substrates are recognised by several members in an enzyme family and, therefore, are equally non-specific. Synergist-insecticide bioassays and biochemical assays are currently recommended follow-up techniques to determine the resistance mechanisms in resistant mosquito populations [17]. These methods have indeed been useful to associate elevated levels of mixed function oxidases with pyrethroid resistance in the field, for example in *An. gambiae* from Kenya [18] and in *An. funestus* from southern Africa [19] but they could not specify which P450s were overexpressed. In an attempt to have more specific assays available, chromo- and fluorogenic substrates with a higher preference for insecticide “metabolisers” have been identified or synthesised in some cases [20, 21], with relatively restricted applicability because of the limited availability of substrates and/or complexity of biochemical reactions.

We believe that multiplex TaqMan assays are the future for resource-efficient and high-throughput monitoring of metabolic resistance in mosquito field populations. Here, we developed novel and rapid multiplex detoxification gene expression assays suitable for monitoring the specific upregulation of genes responsible for metabolic resistance in *Anopheles* mosquitoes. These qPCR assays were designed in the framework of the interdisciplinary research project DMC-MALVEC (dmc-malvec.eu) with the aim to develop an automated diagnostic platform (LabDisk) for malaria vectors [22], though they can also be applied independently in conventionally prepared samples.

## Methods

### Mosquito samples: laboratory colonies and field-caught populations

Non-blood-fed *An. gambiae* female mosquitoes were preserved in RNA*later*® 3–5 days post-eclosion. The following insecticide resistant strains were obtained through BEI Resources, NIAID, NIH: strain AKRON, bulk frozen, MRA-913B, contributed by Martin Akogbeto; strain RSP, bulk frozen, MRA-334 and the strain ZANU MRA-594, both contributed by Hilary Ranson and Frank H. Collins. The VK7 and Tiassalé strains were kindly provided by the Liverpool Insect Testing Establishment (LITE). The AKRON (MRA-913) strain carries the L1014F *kdr* and G119S *Ace-1* target site mutations which leads to phenotypic resistance to carbamate [23]. The RSP (MRA-334) strain’s name stands for reduced susceptibility to permethrin, which is caused by the L1014S *kdr* mutation and increased cytochrome P450 and beta-esterase activity [24, 25]. The metabolic resistance to DDT of the ZANU (MRA-594) strain is conferred by elevated glutathionine-S-transferase and beta-esterase activity [26]. The *An. gambiae* strain from Tiassalé in Côte d’Ivoire exhibits the L1014F *kdr* and G119S *Ace-1* mutations as well as upregulation of several P450s [3, 27]. The combination of these different resistance mechanisms leads to multiple-insecticide resistance to pyrethroids (documented for permethrin and deltamethrin), organochlorides (DDT), carbamates (bendiocarb) and organophosphates (fenitrothion) in the Tiassalé strain. The VK7 strain’s high resistance to pyrethroids and DDT is a consequence of mutations in the common target site of these insecticides. The effect of the fixed L1014F *kdr* mutation is enhanced by the N1575Y super-*kdr* mutation in a substantial proportion of the VK7 colony [28]. The Kisumu and Ngusso laboratory colonies are susceptible to all above mentioned insecticides and were used as control comparator strains in this study. The insectary at the Swiss Tropical and Public Health Institute provided specimens of the Kisumu colony that originates from an MRA-762 egg batch provided by BEI Resources [29]. The Ngusso specimens used in this study were reared in the insectary of the Institute of Molecular Biology and Biotechnology, Foundation for Research and Technology-Hellas. The description of the characteristics of the laboratory colonies included in the study is summarised in Additional file 1: Table S1. Specimens from a field-caught population from Bioko Island that was recently characterised [10] were also included in the validation of the assays.

### Nucleic acid extractions and preparation of samples omitting the extraction step

Mosquitoes (10 individuals per sample) were mechanically disrupted using a tissue grinder and pestle in a 1.5 ml microcentrifuge tube with 200 μl TE buffer (10 mM Tris-HCl, 1mM EDTA, pH 8.0). Nucleic acids (total RNA and DNA) were extracted using the MagnaMedics magnetic-bead based protocol (MagnaMedics GmbH, Aachen, Germany). In brief, 150 μl lysis buffer were added to the previously processed mosquitoes, followed by 10 min incubation at room temperature and a centrifugation step at 16,000× *g* for 2 min in order to sediment non-lysed tissue debris. The clear lysate supernatant was incubated subsequently with 30 μl magnetic beads and 440 μl binding buffer for 10 min and was washed twice with 200 μl wash buffer for 1 min. Nucleic acid elution was performed with 150 μl elution buffer for 10 min at 50 °C. For the direct PCR approach, the clear lysate was directly diluted 25× with DEPC-treated water and used as a template for the RT-qPCR reactions. Smaller dilutions were also tested but the 25× dilution was selected due to both absence of inhibition and optimal sensitivity. Nucleic acid integrity was assessed *via* agarose gel electrophoresis (1.2% w/v).

### Reverse transcription and singleplex qPCR based on SYBR Green chemistry

As a reference method to measure gene expression, singleplex qPCR assays based on SYBR Green chemistry were performed. cDNA was synthesized using 1 μg οf total RNA, previously treated with TURBO™ DNase (Invitrogen, Carlsbad, CA, USA), with oligo (dT)12-18 primers and the Thermoscript RT-PCR system kit (Invitrogen, Carlsbad, CA, USA), following the manufacturer’s instructions. The SYBR Green-based qPCR assays were run in duplicates in 10 μl reactions, consisting of 2× Kapa SYBR® Fast Universal qPCR Master Mix (Kapa Biosystems, Wilmington, MA, USA), forward and reverse primers specific for each gene (Additional file 1: Table S2) at a final concentration of 200 nM as well as 20 ng of cDNA template. Bio-Rad CFX Connect™ Real-Time PCR Detection was used with a thermal protocol consisting of a 3 min polymerase activation/initial denaturation step at 95 °C, 40 cycles of denaturation and annealing/extension steps at 95 °C for 3 s, 60 °C for 30 s, followed by a melting curve analysis step. A no-template control was included in each qPCR run.

### Design of multiplex detox assays

A total of four triplex detox assays [Detox (A)-Detox (D)] were designed using the three fluorophores, FAM (green), HEX (yellow) and Atto647N (red) of the TaqMan probe chemistry. Along with the 40S ribosomal protein S7 (*RPS7*; AGAP010592), *CYP6P3* (AGAP002865) and *CYP6M2* (AGAP008212) compiled Detox (A), *CYP9K1* (AGAP000818) and *CYP6P4* (AGAP002867) Detox (B), *CYP6Z1* (AGAP008219) and *GSTE2* (AGAP009194) Detox (C) and *CYP6P1* (AGAP002868) and *CYP4G16* (AGAP001076) Detox (D). The *RPS7* target has previously shown to be a suitable reference in *An. gambiae* [5, 6] and was included in all assays to normalise each reaction for variations in RNA concentrations.

Primers and probes for the multiplex TaqMan qPCR assays were designed *de novo* (Additional file 1: Table S2). In addition to the standard guidelines for qPCR assays the following criteria were considered: (i) at least one primer was chosen to bridge the junction of two exons to avoid DNA amplification (Additional file 2: Figure S1); (ii) specificity for each gene obtained both by amplification (primers) and detection (probe) properties; and (iii) avoiding cross-reactions with other targets in the same multiplex.

Inclusivity was assessed *in silico* to ensure that the newly developed triplex assays detect all sequences of the target genes in *An. gambiae*. All available sequences for each of the nine target genes were downloaded from the NCBI Nucleotide database and analysed for matches with the corresponding assay using the “Test with saved primers” tool in Geneious 10.2.5 (Biomatters Ltd, Auckland, New Zealand) [30].

In order to assess the exclusivity in terms of sequences and the sibling species of the *An. gambiae* complex, the primers and probes included in the assays from each multiplex were analysed *in silico*. The Perl script Simulate_PCR [31] was used together with BLAST+ 2.7.1 [32]. Briefly, a list with all primers and probes was used to perform a BLAST search against two different databases: the nt database (downloaded from the NCBI BLAST ftp site, version from 08/06/2018) and a list with all DNA sequences available for mosquitoes of the *An. gambiae* species complex in VectorBase [33]. The Simulate_PCR script analyses the BLAST output to look for pairs of primers that could produce an amplicon and only oligos with 4 or fewer mismatches with the target sequence were considered. The amplicons were also only considered if the size was between 40 bp and 500 bp. With the software it was also checked that a probe bound within the potential amplicons.

Primers were also analysed with the software Autodimer-1 [34] for the potential formation of primer dimers between those pairs present in the same multiplex assay. The software calculates a score based on the number of matches (+1) and mismatches (-1) between 2 oligos. The recommended threshold of 7 for this score was used without any potential dimer found. Further tests were performed with more liberal conditions and the most stable of the interactions identified had a T_m_ of 29.7 °C and a deltaG of -3.96 kcal/mole. These interactions were deemed not relevant as the lowest temperature used during the qPCR is 60 °C and the regular threshold for deltaG on primer dimers is -5 kcal/mole.

### Multiplex RT-qPCR Taqman assays

All oligos were optimised in terms of reaction efficiency and sensitivity by prioritising on those oligos that yielded an early Ct value at the lowest concentration. To facilitate the selection process, the reactions with combinations of oligos at different concentrations were carried out and plotted in a heat diagram (Additional file 2: Figure S2) and the pairs that gave maximum yield with the lowest concentrations were chosen. After primer optimisation a range of probe concentrations were also tested following the same criterion as well as fluorescence signal strength. To verify the absence of crosstalk and background signal, each target was amplified individually using corresponding plasmid controls. Additionally, all assays were run comparatively on the same plate in triplex and singleplex formats. The one-step reverse transcription qPCR (RT-qPCR) mastermix that was used in this study was supplied by Fast Track Diagnostics (Esch-sur-Alzette, Luxembourg). Nucleic acids of at least 100 ng per sample were used in a total reaction volume of 10 μl with the primer-probe concentrations given in Additional file 1: Table S2. The thermal cycle parameters were: 50 °C for 15 min, 95 °C for 3 min, and 40 cycles of 95 °C for 3 s and 60 °C for 30 s. The reactions were performed in 96-well plates in a ViiA 7 Real-Time PCR System (Applied Biosystems, Waltham, MA, USA). Samples were amplified in duplicates and each run always included a non-template control.

The sensitivity and specificity of the RT-qPCR assays developed were evaluated through quality control procedures consisting of: (i) the construction of standard curves for all genes both in singleplex and in multiplex formats; (ii) including a control sample consisting of RNase treated template to ensure only mRNA and no gDNA is amplified; (iii) visualisation of the amplicons by agarose gel electrophoresis for all amplicons to check for the presence of a unique band with the expected length of each amplicon’s mRNA (Additional file 2: Figure S3); and (iv) estimating assay reproducibility, expressed by the coefficient of variation (CV), by analysing a series of samples within the same and different runs. The analytical parameters of the RT-qPCR reactions are presented in details in Table 1.

**Table 1:** Quality control characteristics of the qPCR reactions

Gene (assay)	% Reaction efficiency (M)	% Reaction efficiency (S)	R^2^ (M)	R^2^ (S)	Dynamic range (M)	Dynamic range (S)	%CV (M)	%CV (S)
*RPS7* (Detox A-D)	104	103	0.999	0.998	16.50–31.00	16.20–31.00	8.89	9.52
*CYP6P3* (Detox A)	107	102	0.999	0.991	25.70–32.92	25.96–33.46	9.99	12.37
*CYP6M2* (Detox A)	109	103	0.998	0.996	26.39–32.97	26.44–34.31	9.64	10.41
*CYP9K1* (Detox B)	105	101	0.994	0.989	20.22–31.67	20.74–32.02	6.50	9.64
*CYP6P4* (Detox B)	109	107	0.996	0.996	25.54–33.94	25.83–34.30	4.46	10.41
*CYP6Z1* (Detox C)	99	95	0.995	0.997	23.37–33.63	23.29–33.29	5.54	9.99
*GSTE2* (Detox C)	100	96.1	0.999	0.998	23.25–33.36	23.62–33.69	12.07	16.03
*CYP6P1* (Detox D)	97	95	0.997	0.996	24.66–34.17	24.87–34.27	10.37	13.55
*CYP4G16* (Detox D)	100	98	0.999	0.998	19.22–30.30	19.52–30.40	9.02	12.24

*Abbreviations*: *M*, multiplex; *S*, singleplex; *CV*, coefficient of variation at the expression units level

Following quality control, the newly-developed multiplex detox assays were used to measure gene expression in laboratory colonies for which data were available, from the literature and/or from publicly available databases for the expression profiles of the genes included in our assays. Akron, a multi-resistant laboratory colony [35], was also used in this analysis. The same susceptible laboratory strain as reported in each published study/database was used for comparison. Additionally, the standard two-step singleplex RT-qPCR with SYBR Green chemistry was performed for each gene and the % difference between the fold changes measured by the multiplex and singleplex approaches was calculated as described in the Bland Altman analysis [36].

### Statistical analysis

Calculation of fold changes and *P*-values were assessed by the method of Pfaffl et al. [37], implemented in the REST 2009 software v2.013 that allows inputting Ct values for controls (susceptible population) and samples (resistant population) for each gene and returning fold changes for the resistant population with 95% confidence intervals (CIs) and *P*-values. The Ct values of normaliser and each target gene for each sample are jointly reallocated to susceptible and resistant groups and the fold changes are calculated on the basis of mean values after 2000 iterations. This statistical model described by Pfaffl et al. (Pair Wise Fixed Reallocation Randomization test©) [37] has the advantage of making no distributional assumptions and at the same time do not suffer a reduction in power relative to standard parametric tests. Graphs were produced using SigmaPlot v12.0 software. For the correlation analysis between expression levels, the latter were calculated as relative quantification (RQ) units (RQ = 2^-ΔCt^, where ΔCt = Ct_target_ - Ct_normaliser_). The level of significance was set at α = 0.05.

## Results

### Control of quality, analytical performance and of multiplex detox assays. Demonstration of expression analysis

The *in silico* inclusivity analysis predicted that all mRNA sequences available for all genes and their isoforms, where applicable, were bound by the corresponding oligonucleotides with either perfect matching or no more than one mismatch in either the reverse or the forward primer. This suggests that all sequences will be detectable with these new assays. Exclusivity analysis showed that only the nine expected targets and no other sequence are amplifiable and detectable with the new triplex assays in *An. gambiae* (*s.l.*). No amplicon with oligonucleotides from different assays is expected and the sequence analysis found no potential primer dimers between the oligos present in the same multiplex mix.

Results from the standard curve analysis showed similar and within the accepted range [38] efficiencies (95–109%) and R^2^ values (0.989–0.999) both for single- and multi-plex reactions for each gene. Assay reproducibility ranged from %CV = 4.46–12.07% for multiplex and %CV = 9.52–16.03% for singleplex reactions. The quality control data are described in detail in Table 1. No detectable signal was obtained from DNA samples, verifying the validity of primer design across junctions (Additional file 2: Figure S1). The presence of a unique band with the expected length of each amplicon’s mRNA was verified and additional specificity was achieved with probe hybridisation specific for each gene (Additional file 2: Figure S3 and Additional file 2: Figure S1).

**Fig. 1:**
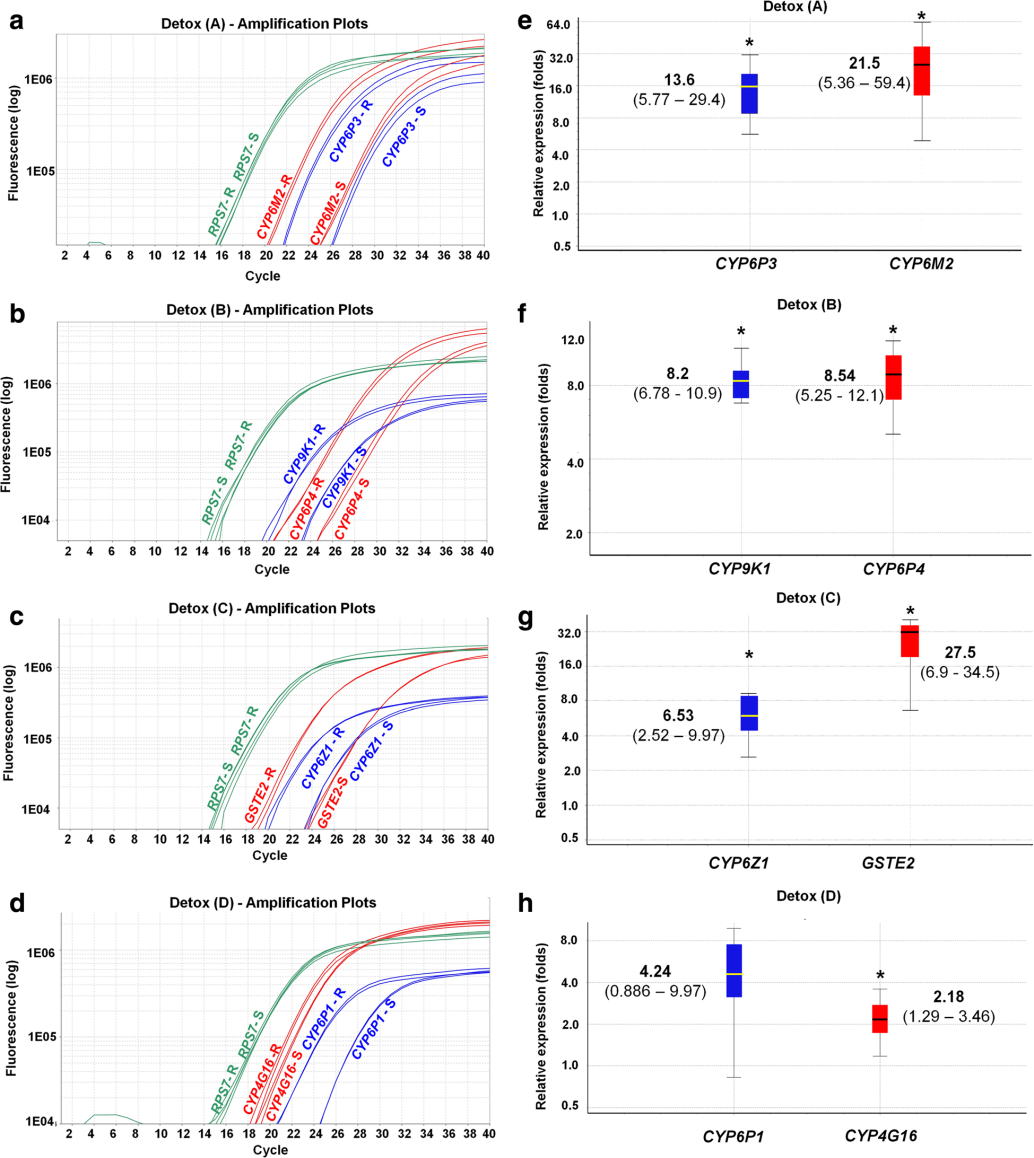
Amplification curves for the four triplex Detox assays (**a**-**d**) and corresponding expression analysis (**e**-**h**) using the REST software. Asterisks indicate statistical significance (*P* ≤ 0.05) (Pfaffl et al. [37]; Pair Wise Fixed Reallocation Randomization test©). *Abbreviations*: R, resistant sample (Akron strain); S, susceptible sample (Kisumu strain)

As an example of the expression analysis output obtained with the new multiplex TaqMan assays, Fig. 1 shows that the multiple-insecticide resistant Akron strain overexpresses all eight detoxification genes compared to the susceptible Kisumu strain. In Fig. 1a-d, the amplification curves of all genes belonging to the four triplex assays are shown; it is clear that for all detoxification genes there are lower Ct values in the resistant compared to the susceptible sample, whereas no change was observed for the *RPS7* reference gene, indicating a clear upregulation of the target (detoxification) genes. In Fig. 1e-h the fold-upregulation for gene is presented along with 95% CIs and *P*-values produced as a simple output by the REST 2009 software.

### Validation of the diagnostic performance of multiplex detox assays using laboratory mosquito colonies

Overall, the newly developed multiplex TaqMan assays produced comparable results with the standard two-step singleplex RT-qPCR method (Table 2). The expression levels defined by the two methods showed a statistically significant correlation for all genes. The overall % difference in fold change determination between the two methods was -17.6%, and no significant bias was detected (95% CI of % difference = -38.7–3.43%).

**Table 2:** Multilevel validation of multiplex detox assays by comparing corresponding expression analysis results to published data and the “gold-standard” singleplex RT-qPCR method. *n* =3 replicates were used for the multiplex detox assays and singleplex RT-qPCR analysis and *P-*values were calculated by the pair wise fixed reallocation randomization test. For the correlation analysis *P-*values were calculated by Pearson’s correlation coefficient (*r*) test

Gene	Samples compared (R *vs* S)	Method			Comparison between multiplex and singleplex RT-qPCR assays	
		Published data	Multiplex detox assays	Singleplex RT-qPCR		
		Fold change (*P-*value) [Reference]	Fold change (95% CI, *P-*value)	Fold change (95% CI, *P-*value)	Mean % difference of fold changes (95% CI)	*r* (*P-*value)
*CYP6P3*	VK7 *vs* NG	3.30 (*P* = 0.013) [42]	3.53 (1.44–8.14) *P* < 0.001	6.58 (1.68–26.39) *P* < 0.001	-13.60 (-127–100)	0.703 (*P* = 0.001)
	TIA *vs* NG	29.5 (*P* = 0.005) [27]	20.7 (9.30–34.2) *P* = 0.033	15.1 (6.83–40.9) *P* < 0.001		
	AKR *vs* KIS	– [23, 35]	13.6 (5.77–29.4) *P* = 0.024	15.3 (6.59–32.4) *P* < 0.001		
*CYP6M2*	VK7 *vs* NG	1.80 (*P* = 0.0044) [42]	6.58 (3.92–8.94) *P* = 0.031	3.73 (1.98–9.48) *P* < 0.001	2.32 (-116–120)	0.941 (*P* < 0.001)
	TIA *vs* NG	6.41 (*P* = 0.015) [27]	4.46 (0.942–21.1) *P* = 0.059	4.99 (0.72–34.6) *P* = 0.103		
	AKR *vs* KIS	– [23, 35]	21.5 (5.36–59.36) *P* = 0.006	31.3 (13.1–99.0) *P* < 0.001		
*CYP9K1*	VK7 *vs* NG	1.40 (*P* = 0.059) [42]	1.28 (0.958–1.71) *P* = 0.094	2.94 (0.942–9.20) *P* = 0.063	-56.47 (-151–38.4)	0.809 (*P* = 0.003)
	TIA *vs* NG	4.33 (*P* = 0.007) [27]	2.01 (1.72–2.32) *P* < 0.001	4.60 (1.80–18.3) *P* < 0.001		
	AKR *vs* KIS	– [23, 35]	8.20 (6.78–10.9) *P* = 0.006	9.28 (2.48–20.9) *P* = 0.026		
*CYP6P4*	VK7 *vs* NG	2.11 (*P* = 0.11) [42]	6.77 (5.20–8.49) *P* = 0.014	8.97 (7.50–10.7) *P* < 0.001	39.65 (-123–202)	0.856 (*P* < 0.001)
	TIA *vs* NG	14.4 (*P* = 0.0047) [27]	13.0 (7.92–18.4) *P* < 0.001	8.33 (4.05–17.6) *P* = 0.033		
	AKR *vs* KIS	– [23, 35]	8.54 (5.25–12.1) *P* < 0.001	2.73 (1.23–6.73) *P* = 0.043		
*CYP6Z1*	RSP *vs* KIS	3.50 (*P* < 0.05) [43]; 1.31 (*P* < 0.05) [44]	3.03 (2.58–3.38) *P* = 0.036	2.69 (1.80–4.79) *P* < 0.001	-35.86 (-101–29.3)	0.512 (*P* = 0.03)
	ZANU *vs* KIS	> 2.0 (*P* < 0.05) [44]	3.27 (1.20–5.23) *P* = 0.045	3.25 (0.81–12.98) *P* = 0.095		
	TIA *vs* NG	1.21 (*P* > 0.05) [27]	2.14 (0.625–4.68) *P* = 0.173	2.52 (0.819–7.76) *P* = 0.11		
	VK7 *vs* NG	1.40 (*P* = 0.013) [42]	0.874 (0.747–1.02) *P* = 0.09	3.31 (1.02–10.75) *P* = 0.050		
	AKR *vs* KIS	– [23, 35]	6.53 (2.52–9.97) *P* = 0.041	12.0 (3.55–77.4) *P* = 0.001		
*GSTE2*	ZANU *vs* KIS	7.80 (*P* < 0.05) [45] 3.90 (*P* < 0.05) [44]	57.8 (52.3–67.5) *P* = 0.041	22.7 (4.24–328.6) *P* < 0.001	20.58 (-50.5–91.7)	0.678 (*P* = 0.003)
	RSP *vs* KIS	2.36 (*P* < 0.05) [44]	30.7 (26.6–34.3) *P* = 0.017	16.5 (1.30–161.3) *P* < 0.001		
	TIA *vs* NG	0.649 (*P* > 0.05) [27]	0.652 (0.406–1.05) *P* = 0.08	0.735 (0.340–1.58) *P* = 0.382		
	VK7 *vs* NG	0.909 (*P* = 0.75) [42]	1.30 (0.891–1.90) *P* = 0.174	1.015 (0.403–2.78) *P* = 0.899		
	AKR *vs* KIS	– [23, 35]	27.5 (6.90–34.5) *P* = 0.034	49.5 (12.5–347.3) *P* < 0.001		
*CYP6P1*	VK7 *vs* NG	2.20 (*P* = 0.048) [42]; 1.70 (*P* < 0.05) [46]	1.55 (1.21–1.84) *P* = 0.024	2.64 (1.75–3.33) *P* < 0.001	-59.84 (-111– -8.5)	0.703 (*P* = 0.002)
	TIA *vs* NG	1.84 (*P* = 0.0037) [27]	2.85 (2.56–3.28) *P* = 0.023	4.48 (2.54–6.91) *P* < 0.001		
	AKR *vs* KIS	– [23, 35]	4.24 (0.886–9.79) *P* = 0.066	10.3 (2.17–44.9) *P* = 0.036		
*CYP4G16*	VK7 *vs* NG	1.90 (*P* < 0.05) [46]; 1.10 (*P* = 0.59) [42]	2.03 (1.67–2.51) *P* = 0.019	2.63 (1.75–3.33) *P* < 0.001	-51.21 (-109–7.1)	0.708 (*P* < 0.001)
	TIA *vs* NG	0.455 (*P* > 0.05) [27]	1.20 (0.760–1.75) *P* = 0.319	2.55 (0.980–10.9) *P* = 0.052		
	AKR *vs* KIS	– [23, 35]	2.18 (1.29–3.46) *P* = 0.023	3.87 (1.03–23.2) *P* = 0.050		

*Abbreviations*: NG, Ngusso strain; TIA, Tiassalé strain; AKR, Akron strain; KIS, Kisumu strain

For each individual gene, a direct comparison of the results obtained from the novel multiplex TaqMan assays, standard singleplex SYBR Green based qPCR and publicly available data is presented in Table 2. *CYP6P3* and *CYP6M2* were upregulated in the Tiassalé *vs* Ngousso comparison in the literature (29.5× and 6.41×), the singleplex RT-qPCR (15.1× and 4.99×) and the multiplex Detox (A) panel (20.7× and 4.46×). Both genes were also upregulated in VK7 compared to Ngousso. *CYP9K1* and *CYP6P4* were upregulated 4.33× and 14.4× in Tiassalé *vs* Ngousso according to the literature, which is consistent with the 2.01× and 13.0× upregulation observed using the Detox (B) panel, and which was further confirmed with singleplex qPCR (4.60× and 8.33× upregulation). Similarly, *CYP6Z1* and *GSTE2* have been previously identified to be significantly overexpressed (3.50- and 7.80-fold) in both RSP and ZANU resistant strains when compared with the susceptible Kisumu strain and this observation is validated both by the newly developed multiplex Detox assay (C) panel (3.03- and 57.8-fold) and the singleplex qPCR (2.69- and 22.7-fold). According to published data *CYP6P1* and *CYP4G16* are marginally upregulated in the resistant VK7 when compared to the susceptible Ngousso strain (2.20- and 1.90-fold, respectively). This marginal difference in gene expression is also observed when assaying the same strains with the Detox assay (D) panel (1.55- and 2.03-fold) and singleplex qPCR (2.64- and 2.63-fold).

Additionally, differences that are not statistically significant according to published data and the standard singleplex RT-qPCR are also concordant with the multiplex detox assays. These negative predictive properties of our method were found for *CYP6Z1*, *GSTE2* and *CYP4G16* in Tiassalé *vs* Ngousso comparisons and *CYP9K1* in VK7 *vs* Ngousso comparison.

### Validation of the diagnostic performance of multiplex detox assays with field-caught specimens

**Fig. 2:**
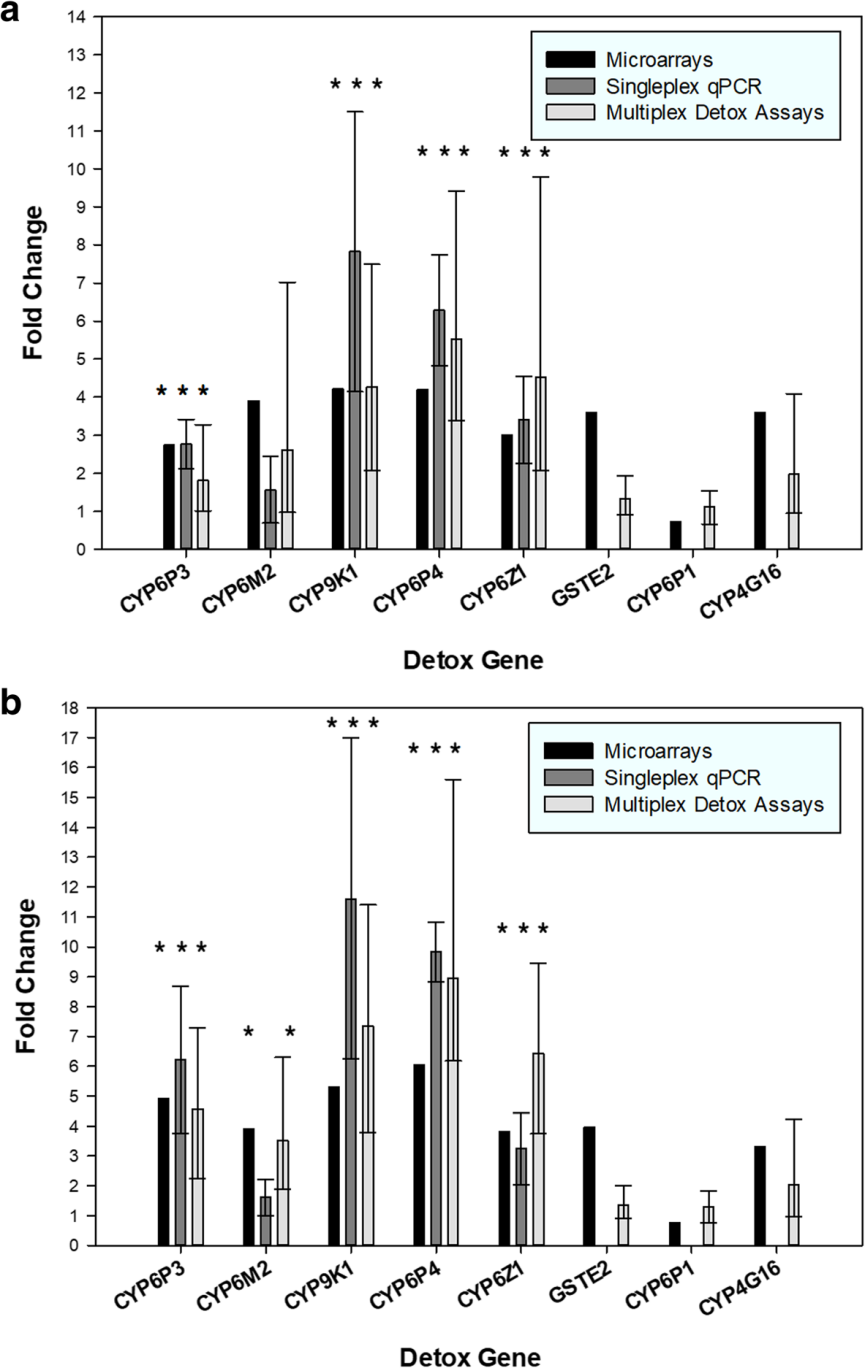
Expression analysis in field-caught samples from Bioko Industrial (**a**) and Hospital (**b**) areas *vs* the Kisumu susceptible strain (*n* = 3 replicates each), showing accordance between the multiplex detox assays and two different methodologies (microarrays and SYBR Green singleplex RT-qPCR). Levels in error bars indicate 95% CIs; asterisks indicate statistical significance (*P* ≤ 0.05)

In addition to the experiments above, RNA templates from two deltamethrin-resistant field populations, hereafter named “Industrial” and “Hospital”, from the Ela Nguema district of Malabo in Bioko (described in detail in [10]) were included in this study. Here, the idea was to further validate the multiplex approach against additional methods, two step singleplex RT-qPCR and DNA microarrays, by measuring the fold changes in expression levels of the eight detoxification genes between the field mosquitoes and a susceptible Kisumu lab colony [10]. Indeed, the estimated fold changes between the two Bioko field populations and the susceptible lab colony were in good agreement between all three methods (Fig. 2). For example, *CYP9K1* showed a 4.21-fold upregulation in the “Industrial” population as shown by the microarrays of the initial study (Fig. 2a). In the two step singleplex RT-qPCR assays the fold change was 7.82 (95% CI: 4.14–11.5) and the current assays yielded a 4.27-fold (95% CI: 2.07–7.49) change. In the Hospital population the microarray output reports a 5.3-fold overexpression for *CYP9K1* (Fig. 2b), which agrees with the 7.35-fold (95% CI: 3.79–11.4) upregulation found with the multiplex detox assays and 11.6-fold (95% CI: 6.25–17.0) in the two step singleplex RT-qPCR method.

Similarly, highly consistent comparative results between the three methods were obtained for *CYP6P3*, *CYP9K1*, *CYP6P4*, *CYP6M2* and *CYP6Z1* for both the Industrial (Fig. 2a) and Hospital (Fig. 2b) field populations. The complete expression analysis results are presented in Additional file 1: Table S3 for the Industrial area and in Additional file 1: Table S4 for the Hospital area population.

For *GSTE2*, *CYP6P1* and *CYP4G16* no singleplex RT-qPCR data are available because they were not followed up in the original study given the microarrays showed no differential expression. These observations are confirmed by our method.

### Ultra-rapid and cost-efficient application of the validated multiplex detox assays with the direct RT-qPCR approach

**Fig. 3:**
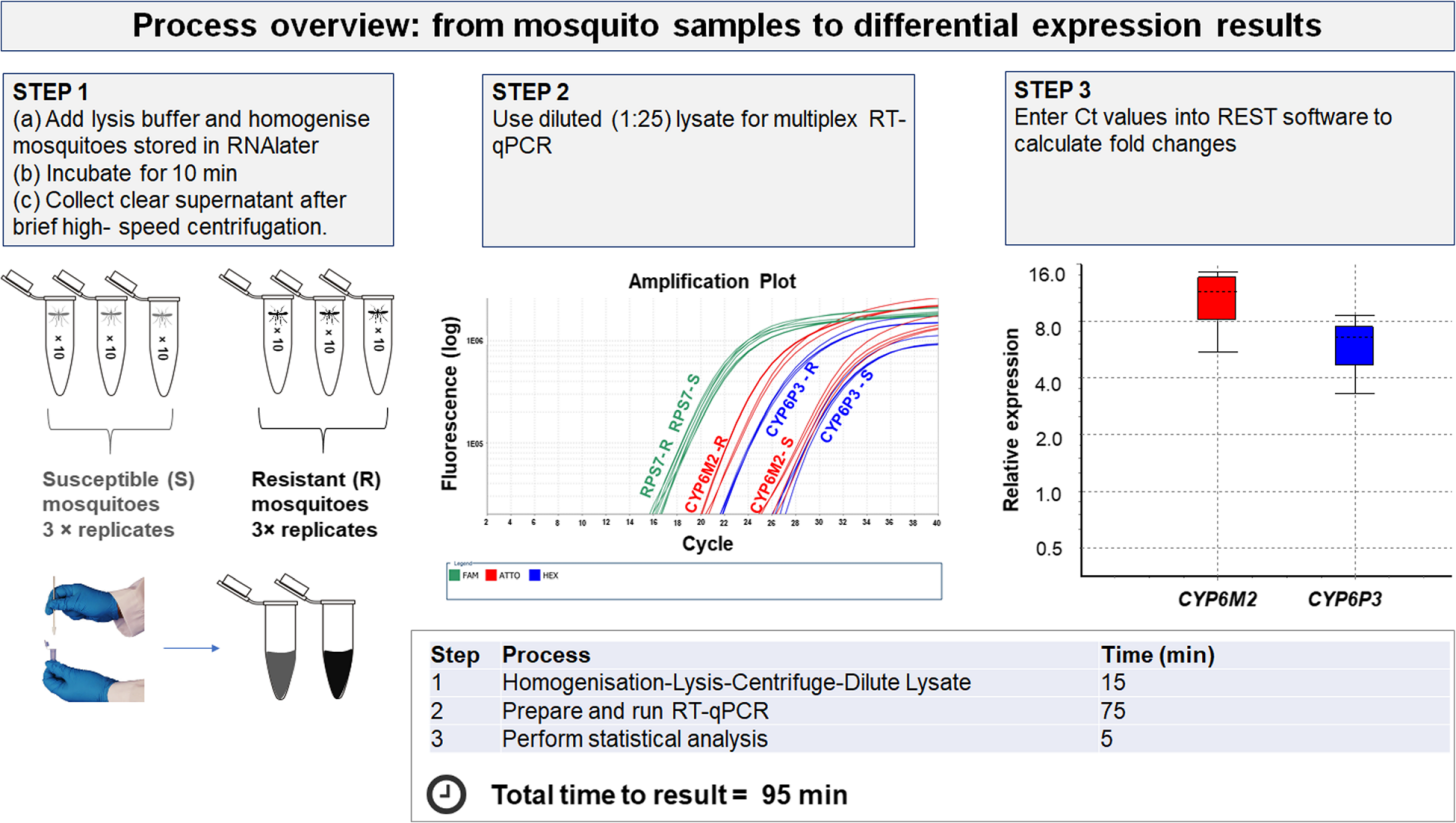
A three-step process from sample lysis to gene expression analysis for two populations (Resistant *vs* Susceptible, *n* = 3 replicates each). For the abovementioned samples a total-time-to-results of approximately 95 min is needed

In order to provide an even more rapid and cost-effective format for analysing detoxification gene expression levels, the possibility of omitting the nucleic acid extraction step was assessed. In order to measure the effect of omitting the extraction step, mosquitoes from the susceptible Kisumu and the resistant Akron colonies were homogenised and lysed before they were divided into two aliquots. Nucleic acids were extracted from the one aliquot, while the other aliquot served directly as a RT-qPCR template after it was further diluted at a 1:25 ratio. Expression analysis data obtained from the aliquot that was directly subjected to RT-qPCR were completely consistent with those obtained from the aliquot from which the nucleic acids were purified. For example, in the comparison between resistant and susceptible mosquitoes using the Detox (A) assay, *CYP6P3* was found to be overexpressed 4.68× (95% CI: 2.67–8.81) with the direct RT-qPCR approach compared with a 4.10-fold overexpression (95% CI: 3.35–5.23) when following the standard nucleic acid extraction procedure. Similarly, the difference for *CYP6M2* was minor between the two approaches (4.57-fold compared to 2.70-fold upregulation for direct RT-qPCR compared to standard procedure, respectively). Detailed results for all genes are presented in Table 3. To corroborate this analysis, gene expression values obtained from the two approaches were also correlated and correlation coefficients indicated a significantly strong correlation (*r* > 0.9 in most cases) (Additional file 1: Table S5). Figure 3 provides an easy-to-follow three-step overview of the direct RT-qPCR procedure from sample-to-result for which only 95 min are needed.

**Table 3:** Gene expression analysis performed in mosquito lysates by direct qPCR and matched purified eluates after nucleic acid extraction (*n* = 4 susceptible *vs n* = 4 resistant). *P* values were calculated by the pair wise fixed reallocation randomization test

Gene (Detox assay)	Direct qPCR in lysates		qPCR in matched nucleic acid extracted eluates	
	Fold change (95% CI)	*P*-value	Fold change (95% CI)	*P*-value
*CYP6P3* (A)	4.68 (2.67–8.81)	0.001	4.10 (3.35–5.23)	0.020
*CYP6M2* (A)	4.57 (3.18–7.19)	0.002	2.70 (2.18–3.40)	0.022
*CYP9K1* (B)	4.66 (3.40–7.70)	0.002	4.45 (4.12–4.80)	<0.001
*CYP6P4* (B)	2.29 (1.31–3.71)	0.018	1.67 (1.33–2.11)	<0.001
*CYP6Z1* (C)	2.98 (2.47–3.84)	0.009	1.85 (1.44–2.22)	<0.001
*GSTE2* (C)	13.6 (9.64–18.4)	0.008	9.36 (7.96–11.9)	<0.001
*CYP6P1* (D)	1.32 (0.968–1.90)	0.060	1.64 (0.752–3.57)	0.215
*CYP4G16* (D)	2.81 (1.61–4.26)	0.008	1.91 (1.45–2.46)	<0.001

## Discussion

We developed a simple, robust, rapid and cost-effective method for determining the expression levels of major detoxification genes that have been associated with metabolic resistance in the malaria vector *An. gambiae*. The assay is based on specific TaqMan probes and RT, and it can reliably determine the expression levels of the pyrethroid metabolisers *CYP6P3*, *CYP6M2*, *CYP9K1*, *CYP6P4*, *CYP6Z1* and *GSTE2*, which have been functionally implicated in metabolic pyrethroid resistance in *An. gambiae*. The assay panel is also readily expandable to capture additional loci as they evolve, or to cover other mosquito vectors.

The method was developed and successfully validated with mosquito specimens from both laboratory colonies and field collections. The results obtained are consistent (both in terms of extent of upregulation and statistical significance) with previously-used methods regarded as gold standards for gene expression determination, such as the two-step standard singleplex qPCR and microarrays, which are far more expensive and complex. The comparative analysis between multiplex TaqMan assays and SYBR Green singleplex qPCR did not reveal significant discordance for the majority of genes (Table 2) with the exception of *CYP6P1* (95% CI of %difference: -111– -8.5, not including the null value). Furthermore, compared to other genes, *CYP9K1* and *CYP4G16* show relatively noticeable differences in fold changes between multiplex and singleplex assays (-56.5% and -51.2% difference, respectively). A possible explanation for this observation could be given by the fact that *CYP6P1*, *CYP4G16* and *CYP9K1* are among the genes that did not show large or statistically significant differences in most of the available comparisons between the lab strains of Table 2, so some variability in determinations between methods is expected. However, even for these genes, available data from the third independent method (microarrays) were in every case in better agreement with the newly developed multiplex, rather than the singleplex assays (Table 2, Additional file 1: Table S3 and Additional file 1: Table S4). All points above suggest that our multiplex assays represent a valid alternative to conventional two-step singleplex qPCR. The “closed-tube” nature of the TaqMan platform means that there is no requirement for post-PCR processing and consequently assays are simple to perform and rapid to run, while the output is easy to produce and interpret. A necessary requirement for these triplex-assay is a real-time PCR machine equipped with three detection channels. Such machines are generally more expensive (approximate price range = 20,000–25,000 €) than machines with only two detection channels (approximate price range = 10,000–15,000 €), but there is currently fast progress in the development of cheap portable field-deployable real-time qPCR thermocyclers. The investment in a flexible multi-channel qPCR machine is worthwhile because multiplex qPCR assays have much lower running cost. In Table 4 we estimated the cost for measuring the gene expression levels of the eight detox genes and one normaliser gene for 12 samples. For the singleplex approach at least 2.25× more mastermix and plastic consumables are needed than for the multiplex assay, while the multiplex method only requires additional TaqMan probes that cost roughly 10 € extra per 96-well plate. The total consumables and reagents for the multiplex TaqMan assay cost approximately half as much as for the same tests with the singleplex SYBR Green assay.

**Table 4:** Cost comparison of triplex TaqMan *vs* singleplex SYBR Green approach. This is a rough cost estimation for measuring the gene expression levels of eight detox genes and one normaliser gene in 12 samples on a standard 96-well plate format

Consumables and reagents	TaqMan triplex RT-qPCR	SYBR Green singleplex RT-qPCR
96-well plastic plate and optical film	1 × 6.50 € = 6.50 €	3 × 6.50 € = 19.50 €
Mastermix	1 × 25.00 € = 25.00 €	2.25 × 25.00 € = 56.25 €
Primers	1 × 0.50 € = 0.50 €	2.25 × 0.50 € = 1.13 €
TaqMan Probes	1 × 10.00 € = 10.00 €	–
Total cost	42.00 €	76.88 €

Furthermore, the presented assays run with mosquito lysates without the requirement of prior RNA extraction and DNase treatment steps due to the primer design across exons. The results suggest that there is little, if any, loss in sensitivity, compared to standard nucleic acid extraction approaches. The extraction-free multiplex RT-qPCR method is easier to perform than the singleplex assays, reliable and rapid, with a total time from sample-to-result as short as 95 min. The addition of the extraction step alone would add an additional 1 h approximately and the performance of assays with the singleplex format would significantly increase the time to result even further (preparation of three reactions instead of one and possibly more than one qPCR run required dependent on the number of samples). The direct-in-lysates RT-qPCR approach seems to produce more variable results (indicated by broader 95% CIs) compared to the standard RNA extraction method and thus more technical replicates might be required to achieve similar precision.

Our method has important features for the future development of diagnostic kits. It does not require a cold chain, as it can be used with samples preserved in RNA*later*® and could be performed with ready-to-use lyophilised RT-PCR enzyme/primer/probe pellets. It is also flexible to include additional genetic traits for insecticide resistance as they evolve and to expand to other species. Furthermore, the format we suggest could include other diagnostic markers such as species identification [39] and *Plasmodium* detection [40] markers. Thus, the multiplex assays described here along with the suggested expansions represent ideal candidates for incorporation in automated diagnostic platforms for mosquito vector surveillance such as the DMC-MALVEC-LabDisk currently under development [22].

## Conclusions

The novel multiplex qRT-PCR assays for monitoring the expression levels of genes previously associated with metabolic resistance in *An. gambiae* (*s.l.*) are simple to perform, robust, rapid, cost-effective and work with RNA*later*® preserved field-collected mosquitoes that can be shipped at room temperature. The approach we suggest here is, therefore, relevant and suitable for insecticide resistance management (IRM) programmes, on the one hand because large numbers of specimens can be handled without extra care, and on the other hand because the speed of the assay allows for producing data as required within short time. The specificity of P450-based metabolic resistance against some but not other active ingredients, as well as the “unexpected case” of cross-resistance against different groups of insecticides [41], indicates that the development of diagnostic tools able to detect specific detoxification enzyme-based resistance is important. Together with bioassays and molecular tests for target site resistance detection, our assays could identify resistance at an early stage and guide efficient and sustainable IRM strategies.

## Additional Files

Additional file 1: **Table S1.** List of laboratory strains and their characteristics. **Table S2.** Primers and probes used in the study. **Table S3.** Expression analysis in field-caught samples from Bioko (Industrial area) *vs* Kisumu susceptible strain. **Table S4.** Expression analysis in field-caught samples from Bioko (Hospital area) *vs* Kisumu susceptible strain. **Table S5.** Accordance between expression levels measured in mosquito lysates by direct qPCR and purified eluates after nucleic acid extraction (*n* = 8) (DOCX 29 kb)

Additional file 2: **Figure S1.** Primer and probe design strategy using Detox (A), consisting of *RPS7* (normaliser), *CYP6P3* (target gene 1), *CYP6M2* (target gene 2) as an example. For each gene either the forward (*RPS7* and *CYP6P3*) or the reverse primer (*CYP6M2*) spanned two exons in order to avoid DNA amplification. Boxes indicate exons, lines indicate introns. *Abbreviations*: F, forward primer; R, reverse primer; P, TaqMan probe labelled with different dyes for each gene; bp, base pairs. **Figure S2.** Results from the primer matrices experiments for each individual gene. Ct values are plotted *versus* forward and reverse primer concentration concentrations. “X” indicates the selected combination of forward and reverse primer concentrations by using as criterion the lowest concentration that gives the earliest Ct values. **Figure S3.** Agarose gel (2.0% w/v) electrophoresis indicating the specificity of the study’s assays. Additional specificity is achieved with probe hybridisation (TaqMan chemistry) (DOCX 3803 kb)

## Abbreviations

CI: Confidence interval
CV: Coefficient of variation
DDT: Dichlordiphenyltrichlorethan
IR: Insecticide resistance
IRM: Insecticide resistance management
RT-qPCR: Reverse transcription qPCR
