# Supplementary material for: Rapid multiplex gene expression assays for monitoring metabolic resistance in the major malaria vector Anopheles gambiae
Source: Parasit Vectors. 2019 Jan 6;12:9. doi: 10.1186/s13071-018-3253-2 (PMC6322220; doi:10.1186/s13071-018-3253-2)
Supplement: Supplementary file 1 — Table S1. List of laboratory strains and their characteristics. Table S2. Primers and probes used in the study. Table S3. Expression analysis in field-caught samples from Bioko (Industrial area) vs Kisumu susceptible strain. Table S4. Expression analysis in field-caught samples from Bioko (Hospital area) vs Kisumu susceptible strain. Table S5. Accordance between expression levels measured in mosquito lysates by direct qPCR and purified eluates after nucleic acid extraction (n = 8) (DOCX 29 kb) [file 13071_2018_3253_MOESM1_ESM.docx]

**Table S1** List of laboratory strains and their characteristics

| **Strain** | **Type** | **Species** | **kdr L1014F**  (% MAF) | **kdr L1014S**  (% MAF) | **kdr N1575Y**  (% MAF) | **iAChe G119S**  (% MAF) | **Metabolic resistance** | **Source (code)** |
| --- | --- | --- | --- | --- | --- | --- | --- | --- |
| Kisumu | S | AG | No (0%) | No (0%) | No (0%) | No (0%) | No | BEI  (MRA-762) |
| Ngousso | S | AC | No (0%) | No (0%) | No (0%) | No (0%) | No | FORTH |
| ZANU | R | AG | No (0%) | No (0%) | No (0%) | No (0%) | Yes | BEI  (MRA-594) |
| RSP | R | AG | No (0%) | Yes (100%) | No (0%) | No (0%) | Yes | BEI  (MRA-334) |
| Akron | R | AC | Yes (100%) | No (0%) | No (0%) | Yes (16.0%) | Yes | BEI  (MRA-913) |
| VK7 | R | AC | Yes (100%) | No (0%) | Yes (40%) | No (0%) | Yes | LITE |
| Tiassalé | R | AG/AC | Yes (95%) | No (0%) | No (0%) | Yes (40%) | Yes | LITE |

*Abbreviations*: AG, *Anopheles gambiae s.s*; AC, *Anopheles coluzzii*; FORTH, Foundation for Research and Technology – Hellas; BEI, BEI resources; LITE, The Liverpool Insect Testing Establishment; MAF, Mutant Allele Frequency; R, Resistant; S, Susceptible.

**Table S2** Primers and probes used in the study

| **Oligo** | **Detox Assay** | **Sequence (5'-3')** | **Concentration (nM)** |
| --- | --- | --- | --- |
| *RPS7*_F | (A)-(D) | CCACCATCGAACACAAAGTTGA | 100 |
| *RPS7*_R | (A)-(D) | TGCTGCAAACTTCGGCTATTC | 200 |
| *RPS7*_P | (A)-(D) | FAM-CCGTGACGTTACGTTCGAATTCCCA-BHQ1 | 250 |
| *CYP6P3*_F | (A) | ACAATGTGATTGACGAAACCCT | 400 |
| *CYP6P3*_R | (A) | GGATCACATGCTTTGTGCCG | 500 |
| *CYP6P3*_P | (A) | HEX-ACCCGCGTACCGTCTGTGGACT-BHQ1 | 350 |
| *CYP6M2*_F | (A) | CTGGCGTTGAATCCAGAGGT | 600 |
| *CYP6M2*_R | (A) | GATACTTGCGCAGTGATTCATTAAG | 400 |
| *CYP6M2*_P | (A) | ATTO647N-AGAGAAATCCTGCAAAAGCACAACGGAGA-BHQ3 | 250 |
| *CYP9K1*_F | (B) | CCGACACGTGGTGATGGATAC | 200 |
| *CYP9K1*_R | (B) | CGTCGTCGGTCCAGTCAAC | 400 |
| *CYP9K1*_P | (B) | HEX-CAATCTTCTGATGCAGGCCCGCAA-BHQ1 | 300 |
| *CYP6P4*_F | (B) | CTGGACAACGTTATCAATGAAACC | 400 |
| *CYP6P4*_R | (B) | GCACGGTGTAATCACGCATC | 500 |
| *CYP6P4*_P | (B) | ATTO647N-CCGATCGAGTCACTTTCGCGCG-BHQ3 | 300 |
| *CYP6Z1*_F | (C) | CCCGCAACTGTATCGGTCTG | 100 |
| *CYP6Z1*_R | (C) | TTCGGTGCCAGTGTGATTGA | 600 |
| *CYP6Z1*_P | (C) | HEX-TGATGCTGTCCCGATTTAACTTTTCGGC-BHQ1 | 250 |
| *GSTE2*_F | (C) | CCGGAATTTGTGAAGCTAAACC | 100 |
| *GSTE2*_R | (C) | GCTTGACGGGGTCTTTCGG | 400 |
| *GSTE2*_P | (C) | ATTO647N-CGGTACGATCATCACCGAGAGCCAC-BHQ3 | 300 |
| *CYP6P1*_F | (D) | ACAGGTGGTGAACGAAACCC | 100 |
| *CYP6P1*_R | (D) | GGTGTAATCCTGTCCCGCAA | 500 |
| *CYP6P1*_P | (D) | HEX-CCGCTCGAAACGACGCTGCG-BHQ1 | 300 |
| *CYP4G16*_F | (D) | GTCCAAGAAGTTGCGTCGGAC | 200 |
| *CYP4G16*_R | (D) | TCTTCGATTTGCGTTGACGTG | 200 |
| *CYP4G16*_P | (D) | ATTO647N-CTGCAGGCCGACATCATTTTGAAGC-BHQ3 | 300 |

*Abbreviations*: F, Forward primer; R, Reverse Primer; P, TaqMan Probe

**Table S3** Expression analysis in field caught samples from Bioko (Industrial area) *vs* Kisumu susceptible strain (N=3 replicates each).

| **GENE** | **Method** | | |
| --- | --- | --- | --- |
|  | Microarrays | Singleplex qPCR | Multiplex Detox assays |
|  | Fold change (*P-*value) | Fold change (95% CI, *P-*value) | Fold change (95% CI, *P-*value) |
| ***CYP6P3*** | **2.74** (*P* < 0.05) | **2.77** (2.12–3.41) *P* < 0.05 | **1.81** (1.01–3.27) *P* = 0.049 |
| ***CYP6M2*** | **3.90** (*P* > 0.05) | **1.56** (0.668–2.45) *P* > 0.05 | **2.61** (0.970–7.02) *P* = 0.057 |
| ***CYP9K1*** | **4.21** (*P* < 0.05) | **7.82** (4.14–11.5) *P* < 0.05 | **4.27** (2.07–7.49) *P* = 0.010 |
| ***CYP6P4*** | **4.19** (*P* < 0.05) | **6.28** (4.82–7.74) *P* < 0.05 | **5.52** (3.38–9.42) *P* < 0.001 |
| ***CYP6Z1*** | **3.00** (*P* < 0.05) | **3.40** (2.26–4.54) *P* < 0.05 | **4.53** (2.08–9.79) *P* = 0.038 |
| ***GSTE2*** | **3.58** (*P* >0.05) | N/A | **1.33** (0.914–1.93) *P* = 0.136 |
| ***CYP6P1*** | **0.725** (*P* > 0.05) | N/A | **1.12** (0.661–1.54) *P* = 0.651 |
| ***CYP4G16*** | **3.6** (*P* > 0.05) | N/A | **1.97** (0.950– 4.08) *P* = 0.068 |

N/A: Not Available

**Table S4** Expression analysis in field caught samples from Bioko (Hospital area) *vs* Kisumu susceptible strain (N=3 replicates each).

| **GENE** | **Method** | | |
| --- | --- | --- | --- |
|  | Microarrays | Singleplex qPCR | Multiplex Detox assays |
|  | Fold change *P*-value (corrected) | Fold change 95% CI, *P*-value | Fold change 95% CI, P value |
| ***CYP6P3*** | **4.90** *P* < 0.05 | **6.22** (3.76–8.67) *P* < 0.05 | **4.57** (2.24–7.29) *P* <0.001 |
| ***CYP6M2*** | **3.90** *P* < 0.05 | **1.63** (0.99–2.22) *P* > 0.05 | **3.52** (1.89–6.29) *P* = 0.016 |
| ***CYP9K1*** | **5.30** *P* < 0.05 | **11.6** (6.25–17.0) *P* < 0.05 | **7.35** (3.79–11.4) *P* <0.001 |
| ***CYP6P4*** | **6.04** *P* < 0.5 | **9.84** (8.84–10.8) *P* < 0.05 | **8.94** (6.18–15.6) *P* < 0.001 |
| ***CYP6Z1*** | **3.81** *P* < 0.05 | **3.24** (2.04–4.44) *P* < 0.05 | **6.42** (3.74–9.45) *P* < 0.001 |
| ***GSTE2*** | **3.95** *P* > 0.05 | N/A | **1.35** (0.903–2.01) *P* = 0.143 |
| ***CYP6P1*** | **0.76** *P* > 0.05 | N/A | **1.30** (0.763–1.82) *P* = 0.368 |
| ***CYP4G16*** | **3.30** *P* > 0.05 | N/A | **2.02** (0.967–4.22) *P* = 0.061 |

N/A: Not Available

| **Gene (Detox assay)** | **r (correlation coefficient)** | ***P-*value** |
| --- | --- | --- |
| *CYP6P3* (A) | 0.969 | <0.001 |
| *CYP6M2* (A) | 0.954 | <0.001 |
| *CYP9K1* (B) | 0.994 | <0.001 |
| *CYP6P4* (B) | 0.947 | <0.001 |
| *CYP6Z1* (C) | 0.933 | 0.001 |
| *GSTE2* (C) | 0.96 | <0.001 |
| *CYP6P1* (D) | 0.619 | 0.102 |
| *CYP4G16* (D) | 0.870 | 0.005 |

**Table S5** Accordance between expression levels measured in mosquito lysates by direct qPCR and purified eluates after nucleic acid extraction (N = 8). P values calculated by Pearson's correlation coefficient test.
