# Supplementary material for: Rapid multiplex gene expression assays for monitoring metabolic resistance in the major malaria vector Anopheles gambiae
Source: Parasit Vectors. 2019 Jan 6;12:9. doi: 10.1186/s13071-018-3253-2 (PMC6322220; doi:10.1186/s13071-018-3253-2)
Supplement: Supplementary file 2 — Figure S1. Primer and probe design strategy using Detox (A), consisting of RPS7 (normaliser), CYP6P3 (target gene 1), CYP6M2 (target gene 2) as an example. For each gene either the forward (RPS7 and CYP6P3) or the reverse primer (CYP6M2) spanned two exons in order to avoid DNA amplification. Boxes indicate exons, lines indicate introns. Abbreviations: F, forward primer; R, reverse primer; P, TaqMan probe labelled with different dyes for each gene; bp, base pairs. Figure S2. Results from the primer matrices experiments for each individual gene. Ct values are plotted versus forward and reverse primer concentration concentrations. “X” indicates the selected combination of forward and reverse primer concentrations by using as criterion the lowest concentration that gives the earliest Ct values. Figure S3. Agarose gel (2.0% w/v) electrophoresis indicating the specificity of the study’s assays. Additional specificity is achieved with probe hybridisation (TaqMan chemistry) (DOCX 3803 kb) [file 13071_2018_3253_MOESM2_ESM.docx]

**Fig. S1** Primer and probe design strategy using Detox (A), consisting of *RPS7* (normaliser), *CYP6P3* (target gene 1), *CYP6M2* (target gene 2) as an example. For each gene either the forward (*RPS7* and *CYP6P3*) or the reverse primer (*CYP6M2*) spanned two exons in order to avoid DNA amplification. Boxes indicate exons, lines indicate introns. *Abbreviations*: F, Forward primer; R, Reverse primer; P, TaqMan probe labelled with different dyes for each gene; bp, base pairs.

**Fig. S2** Results from the primer matrices experiments for each individual gene. Ct values are plotted versus Forward and Reverse primer concentration concentrations. “**X**” indicates the selected combination of forward and reverse primer concentrations by using as criterion the lowest concentration that gives the earliest Ct values.

 **Fig. S3** Agarose gel (2.0% w/v) electrophoresis indicating the specificity of the study’s assays. Additional specificity is achieved with probe hybridisation (TaqMan chemistry).
